# The development of retro-cue benefits with extensive practice: Implications for capacity estimation and attentional states in visual working memory

**DOI:** 10.3758/s13421-021-01138-5

**Published:** 2021-02-22

**Authors:** Paul Zerr, Surya Gayet, Floris van den Esschert, Mitchel Kappen, Zoril Olah, Stefan Van der Stigchel

**Affiliations:** 1 Experimental Psychology, Helmholtz Institute, Utrecht University, Utrecht, The Netherlands; 2 Donders Institute for Brain, Cognition and Behaviour, Radboud University, Nijmegen, The Netherlands

**Keywords:** Visual working memory, Working memory capacity, Sensory memory, Attention, Retro-cues, Change-detection

## Abstract

Accessing the contents of visual short-term memory (VSTM) is compromised by information bottlenecks and visual interference between memorization and recall. Retro-cues, displayed after the offset of a memory stimulus and prior to the onset of a probe stimulus, indicate the test item and improve performance in VSTM tasks. It has been proposed that retro-cues aid recall by transferring information from a high-capacity memory store into visual working memory (multiple-store hypothesis). Alternatively, retro-cues could aid recall by redistributing memory resources within the same (low-capacity) working memory store (single-store hypothesis). If retro-cues provide access to a memory store with a capacity exceeding the set size, then, given sufficient training in the use of the retro-cue, near-ceiling performance should be observed. To test this prediction, 10 observers each performed 12 hours across 8 sessions in a retro-cue change-detection task (40,000+ trials total). The results provided clear support for the single-store hypothesis: retro-cue benefits (difference between a condition with and without retro-cues) emerged after a few hundred trials and then remained constant throughout the testing sessions, consistently improving performance by two items, rather than reaching ceiling performance. Surprisingly, we also observed a general increase in performance throughout the experiment in conditions with and without retro-cues, calling into question the generalizability of change-detection tasks in assessing working memory capacity as a stable trait of an observer (data and materials are available at osf.io/9xr82 and github.com/paulzerr/retrocues). In summary, the present findings suggest that retro-cues increase capacity estimates by redistributing memory resources across memoranda within a low-capacity working memory store.

Subjective perceptual experience suggests that the human visual system can represent many objects simultaneously. When visual input is no longer available, observers can maintain multiple objects in visual short-term memory (VSTM), which traditionally comprises visual working memory (VWM) and sensory memory (Averbach & Coriell, 1961; Baddeley & Hitch, 1974; Sperling, 1960; see Table 1). A more recent conceptualization describes three states in VSTM: “activated” long-term memory (LTM; see Table 1), a large capacity store, (2) a capacity-limited, attended “region of direct access” (VWM), and (3) a strongly attended, single item in the direct focus of attention (Oberauer, 2002; Oberauer & Hein, 2012).

**Table 1:** Names, abbreviations, and definitions of (hypothesized) short-term memory stores discussed in this article

Term	Definition
Visual short-term memory (VSTM)	An overarching term, describing any combination of memory systems that allow for maintaining visual information for a short amount of time.
Sensory memory	A short-lived (100 ms to 2 s), highly fragile, high-capacity buffer of visual information, which is unattended and not available for direct report but contains cueable (bound) objects.
Iconic memory	Sensory memory as described by Sperling (1960). Extremely short lifetime (up to 500 ms) and near-unlimited capacity.
Fragile memory	Sensory memory as described by Sligte, Scholte, and Lamme (2008). Moderate lifetime (up to 2 s) and moderate capacity (16 items).
Visual working memory (VWM)	A highly capacity-limited memory system, which relies on a limited resource (i.e., attention).
Long-term memory (LTM)	Unattended, near-unlimited capacity storage system. According to Oberauer (2002), some portion of it (activated LTM) can supplement working memory performance.

*Note.* The definitions provided in this glossary are our best attempt of paraphrasing the current status quo in the literature regarding these different memory stores. Whereas some of these memory stores have received considerable empirical support, it should be noted that the existence of others is not (yet) established.

While the structure and substrates of these memory systems are not yet fully understood, VWM is generally recognized as highly limited in capacity (e.g., “the magic number four”; Cowan, 2001; Luck & Vogel, 1997). Optimal operation of such a resource-constrained system requires a flexible allocation of a limited memory resource (i.e., attention) to prioritize task-relevant items at the expense of task-irrelevant items. Here, we define “attention” as a cognitive resource that can be deployed across a very limited amount of information simultaneously. As a result, items in VWM are not always homogenously represented. Attention and VWM are closely related and considered to represent a common neural resource (Awh & Jonides, 2001; Cowan, 2001; LaBar, Gitelman, Parrish, & Mesulam, 1999; Mayer et al., 2007). When studied at low set sizes, attended memory items differ from unattended (or less attended) memory items in several ways. Attended, but not unattended, items appear to be maintained in sensory cortices in addition to parietal and frontal areas (Christophel, Iamshchinina, Yan, Allefeld, & Haynes, 2018; Sahan, Sheldon, & Postle, 2019), interact with incoming sensory information (van Loon, Olmos-Solis, & Olivers, 2017; van Moorselaar, Olivers, Theeuwes, Lamme, & Sligte, 2015), and are maintained through persistent neural activity (i.e., sustained firing; Manohar, Zokaei, Fallon, Vogels, & Husain, 2019; see also Stokes, 2015; Wolff, Ding, Myers, & Stokes, 2015; Wolff, Jochim, Akyürek, & Stokes, 2017). Importantly, unattended items are more susceptible to perceptual interference than attended items (i.e., they are more likely to be erased by incoming sensory information; Makovski & Jiang, 2007; Makovski, Watson, Koutstaal, & Jiang, 2010; Matsukura, Luck, & Vecera, 2007; Pinto, Sligte, Shapiro, & Lamme, 2013; Souza, Rerko, & Oberauer, 2014). It has been proposed that attended and unattended items in VWM are represented in a qualitatively different state, such as neural spiking activity-based versus activity-silent working memory storage (Stokes, 2015; Wolff et al., 2015; Wolff et al., 2017). Others have argued that the difference between attended and unattended items is merely quantitative, with attended and unattended items both reflecting activity-based storage (e.g., Rademaker & Serences, 2017; Schneegans & Bays, 2017). Common to all views, however, is that items within VWM are not represented homogenously, as different amounts of attentional resources can be allocated to different items.

It is widely accepted that observers can flexibly reallocate attentional resources within VWM, to prioritize task-relevant objects for recall and cognitive manipulation. Due to the inherent capacity-limitation of VWM, it would be beneficial if observers could also access latent items in separate large-capacity memory stores, from which information can be retrieved by retrospectively attending and transferring items to accessible VWM. Both LTM and sensory memory would represent candidates for such a qualitatively different, large-capacity memory store, as these do not have the same resource limitation as VWM (i.e., attention). It remains unknown, however, whether retrospective allocation of attention allows observers to retrieve items from separate high-capacity memory stores into VWM.

The retro-cue paradigm (Griffin & Nobre, 2003; Landman, Spekreijse, & Lamme, 2003) emerged as a powerful experimental tool to study how an unattended item that was initially stored in a weak state can later be reprioritized (i.e., attended; de Vries, van Driel, Karacaoglu, & Olivers, 2018; Larocque, Riggall, Emrich, & Postle, 2017; Lewis-Peacock, Drysdale, Oberauer, & Postle, 2012; Sprague, Ester, & Serences, 2016; van Loon, Olmos-Solis, Fahrenfort, & Olivers, 2018). A retro-cue is presented after offset of the to-be-memorized stimuli and predicts which item will be tested in the upcoming memory task. This cue allows observers to retrospectively prioritize an item in memory, increasing its likelihood of recall compared with a post-cue condition, in which the test item is designated at the onset of the memory task display. Importantly, retro-cue paradigms, as well as partial-report paradigms, demonstrate that more information is stored in memory than is readily available for report in typical change-detection paradigms.

Sperling’s partial-report experiments already established that more items can be reported if the subset of items that will be tested is made known during the retention interval. A distinction emerged between sensory memory as a high-capacity, unstable, short-lived, and implicit memory system (i.e., iconic memory and informational persistence; Coltheart, 1980; Pratte, 2018) and VWM as a strongly capacity limited, but stable and reportable memory system. Strikingly, the retro-cue paradigm and the partial-report paradigm are conceptually very similar, as both improve memory report by presenting a cue prior to the memory probe. However, retro-cue effects are widely assumed to operate within VWM (for a review of contemporary explanations of the retro-cue effect see, Souza & Oberauer, 2016). On the other hand, Sligte et al. (2008) proposed that retro-cues can access a high-capacity VSTM store that is distinct from VWM: fragile memory. They described fragile memory as a form of sensory memory (see Table 1), characterized by a high capacity, a lifetime of several seconds, high susceptibility to visual interference (Pinto et al., 2013), and not being limited by the amount of attention available for distribution among memory items (Vandenbroucke, Sligte, & Lamme, 2011). In this view, the visual system maintains a high-capacity buffer of location-feature bound objects (Pinto et al., 2013), which can be brought into more stable VWM for cognitive manipulation and retrieval (Sligte, Wokke, Tesselaar, Scholte, & Lamme, 2011; Vandenbroucke et al., 2011; Vandenbroucke, Sligte, de Vries, Cohen, & Lamme, 2015). The retro-cue paradigm would then, in the same way as partial-report cues, constitute a potent way of experimentally inciting participants to transfer items that were initially stored in an unreportable state into VWM for recall and manipulation. Both sensory memory and LTM would fit the description of a large-capacity memory system that is not limited by the same memory resource as VWM. In this study, we test the hypothesis that retro-cues are able to access information in a separate high-capacity store, such as activated LTM (i.e., Oberauer, 2002), or sensory memory (i.e., Sligte et al., 2008).

The view that retro-cues access a separate high-capacity memory store predicts that an observer’s VSTM capacity is mostly limited by their proficiency in utilizing the retro-cue, and not by the lower capacity limits of VWM (see Fig. 1). Indeed, when presenting observers with set sizes far above typical VWM capacity in a retro-cue change-detection paradigm, Sligte et al. (2008) observed dramatically high-capacity estimates. In the condition with the largest set size, observers were, on average, able to report about 16 items from a memory array of 32 items, which is remarkably higher than the typical three-to-four item limit associated with VWM (e.g., Luck & Vogel, 1997). In contrast, Cowan’s *k* (Cowan, 2001), a measure of working memory capacity, remained stable at around four items in the post-cue condition, irrespective of set size. They discovered that *k* scaled with set size in the retro-cue condition. It should be noted that *k* intends to provide a measure of working memory capacity that does not scale with set size (e.g., Rouder, Morey, Morey, & Cowan, 2011), which is the case in change-detection paradigms without retro-cues. Sligte et al. (2008) regarded the observed high-capacity estimates as evidence for the existence of a high-capacity sensory memory store. It is indeed an intriguing hypothesis that the visual system would be able to retrieve information from a memory system (i.e., sensory memory or LTM) that is not subject to the resource limitations of VWM (i.e., attention). Indeed, in line with the view that observers can access more information in visual memory than estimated in typical working memory tasks, other researchers found evidence that observers were able to retain and access 30 images in a rapid serial visual presentation paradigm (Endress & Potter, 2014).

**Fig. 1:**
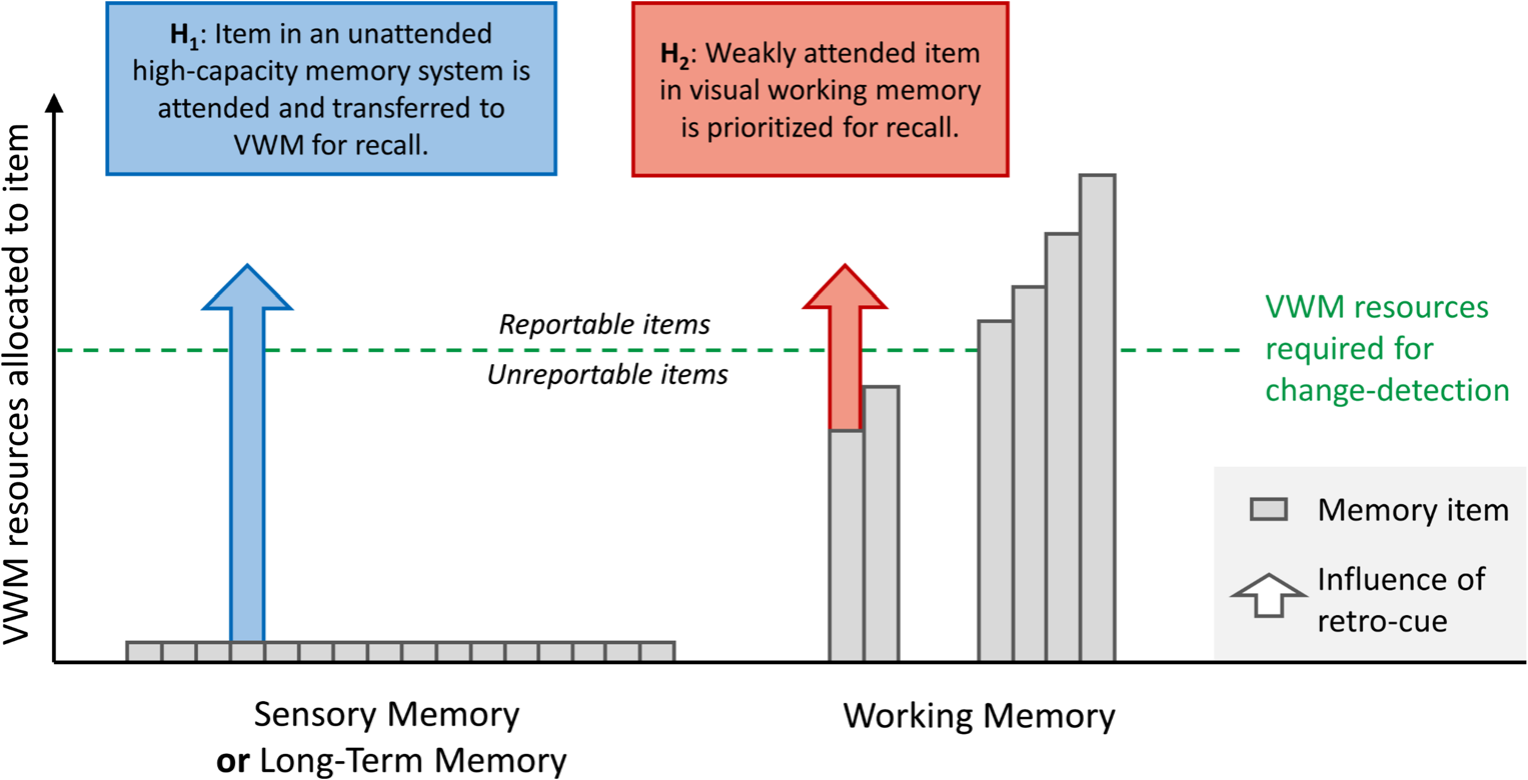
Graphical representation of the investigated hypotheses, which depicts two different ways in which information could be maintained in the visual system, and how reallocation of VWM resources following a retro-cue could act on these representations to improve performance in a change-detection task. The *y*-axis represents the amount of attention (i.e., the memory resource underlying VWM) an item received. The bars on the left represent memory items in sensory memory or LTM, which can be cued (and accessed) with a retro-cue, but which are not subject to the same resource limitation as VWM. The bars on the right represent weakly and strongly attended items represented in low-capacity VWM. The green, dashed line represents the amount of VWM resources required for a memory object to be reportable in a change-detection task. A weakly attended item in VWM would not be reportable due to visual interference by the memory probe. The arrows represent how retro-cues would act on the different memory representations and increase capacity estimations under the multiple-store hypothesis (H_1_; blue arrow) and single-store hypothesis (H_2_; red arrow). (Color figure online)

The interpretation that the retro-cue effect reflects access to a high-capacity memory system has been questioned by some researchers (Makovski, 2012; Matsukura & Hollingworth, 2011; Robinson & Irwin, 2019). There are several competing (and partially complementary) hypotheses to explain the retro-cue effect (Souza & Oberauer, 2016), most prominently, a shift of attention within VWM representations to protect items from visual interference. Matsukura and Hollingworth (2011) argued that the high capacities observed by Sligte et al. (2008) are explained by a long practice session preceding the experiment. They suggested that changes in processes unrelated to memory capacity (e.g., the efficiency of perceptual processing, memory encoding, maintenance, comparison processes, or involvement of long-term memory could account for an increase in performance in memory tasks). In our experience (e.g., Zerr et al., 2017), it indeed typically takes many trials for a retro-cue benefit (the difference between retro-cue and post-cue condition performance) to emerge. Importantly, however, while performance in any task is expected to increase with practice, participants in the study by Sligte et al. (2008) performed just as many trials in the post-cue condition as in the retro-cue condition, yet these high-capacity estimates were only observed in the retro-cue condition. Practice effects that increase proficiency in, for example, the encoding of the memory array or the parsing of the probe array should similarly affect performance in the post-cue and retro-cue conditions. Instead, in order to differentially affect performance in the two conditions, practice effects should specifically improve participants' ability to utilize retro-cues. Moreover, the observation that extensive practice is required for a retro-cue benefit to emerge does not exclude the possibility that retro-cues provide access to a high-capacity memory store: While internal shifts of attention are commonplace in real life, a retro-cue that allows for manipulating these internal shifts of attention experimentally (Griffin & Nobre, 2003; Landman et al., 2003) is an unnatural stimulus, which requires extensive practice to be optimally used. The specific aspects of the paradigm that are impacted by practice, however, depend on whether or not retro-cues allow observers to access a high-capacity memory store that is distinct from VWM. Accordingly, differences between the learning curves in the post-cue and retro-cue conditions may be informative of potential qualitative differences between memory stores that are accessed by shifts of attention.

Studies using the retro-cue paradigm (including Sligte et al., 2008), typically report results as an average collapsed across time. It therefore remains unclear whether learning to use the retro-cue is a slow or rapid process, whether the retro-cue benefit continues to increase indefinitely with more practice, and whether training increases performance in both conditions simultaneously. Observers in Sligte et al. (2008) reached ceiling performance in some set size conditions (four and eight), but not others (16 and 32). It is unclear what caused this pattern of results and whether observers could have reached ceiling performance in all set sizes given enough practice. In the current study, we measured memory capacity estimates throughout an extensive training period, to distinguish between the patterns of training effects that would provide evidence for or against the hypothesis that retro-cues access a high-capacity memory store. We consider two scenarios describing how extensive training might affect memory performance in the post-cue and retro-cue conditions over time (see also Fig. 1):

- H_1_: Retro-cues access a mechanistically and functionally distinct high-capacity memory store (i.e., sensory memory or LTM), which is not subject to the attention-based capacity limitations of VWM. With practice, observers get better at utilizing the retro-cue and bring items into robust VWM for retrieval. This scenario predicts that performance in the retro-cue condition increases continuously (relative to the post-cue condition) until capacity estimates are reached that far exceed traditional VWM capacity (i.e., multiples thereof). Traditional VWM capacity is reflected in performance in the post-cue condition. If the capacity of a memory system that can be accessed through a retro-cue exceeds the set size, then, given enough practice, all (or most) items should become reportable.
- H_2_: Retro-cues operate within a single memory store (VWM) and with practice, observers get better at using the retro-cue to redistribute attentional resources and prioritize less attended items in VWM and increase the probability of retrieval. This scenario predicts that after its initial emergence, the retro-cue benefit stabilizes once the limited capacity of VWM, including less prioritized items, is reached. This capacity estimate would be expected to be not much larger than what is observed in the post-cue condition and certainly smaller than the current set size of 12 items.

We investigated how memory performance in the post-cue and retro-cue conditions develops over time to discern between the two scenarios described above. To this end, 10 observers performed a retro-cue change-detection task with a set size of 12 items for 12 hours over the course of eight sessions. This also ensured that observers reached the maximum retro-cue benefit they could achieve.

## Method

### Participants

Ten Utrecht University students (seven females; ages 19–36 years) took part in the experiment. This is the same number of participants that were tested in Experiment 1 in Sligte et al. (2008). All reported normal or corrected-to-normal vision and no psychiatric diagnosis. Participants gave informed consent, and the study was approved by the local ethics committee. Participants received financial compensation or participant hours (course credits) for their time.

### Setup and stimuli

Stimuli were presented in a dark room on an ASUS PG278q LCD monitor with a display area of 60 × 34 cm and a resolution of 2,560 × 1,440 px at a refresh rate of 100 Hz and response time of 1 ms. Eye movements were monocularly recorded at 1000 Hz on an EyeLink1000 eye tracker (SR Research Ltd, Canada). Participants were seated on an adjustable chair with their head placed on a chin rest 65 cm in front of the screen. Stimuli were presented in MATLAB (2015a) and Psychtoolbox 3 (Brainard, 1997; Kleiner, Brainard, & Pelli, 2007; Pelli, 1997).

**Fig. 2:**
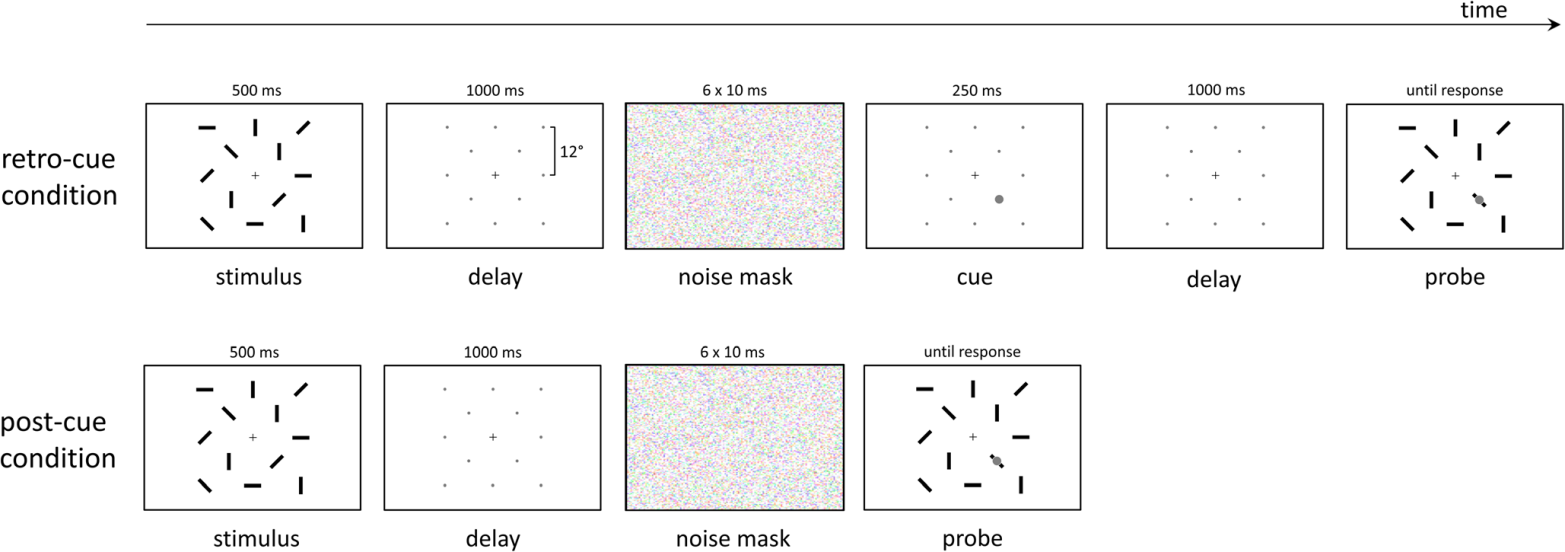
Trial layout. Sizes and colors are stylized in favor of visualization. First, a memory stimulus is displayed, consisting of 12 bars in one of four orientations. After a delay period, during which only the fixation dot and 12 placeholder dots are visible, a noise mask is presented. In the retro-cue condition, a cue in the form of a small circle is then shown on one of the placeholder dots, which indicates the location of the upcoming target to be recalled. This is followed by a delay period and a memory probe display, consisting of the same 12 oriented bars, one of which has a 50% chance of being rotated by 90°. This target is indicated by a small circle, identical to the retro-cue. In the post-cue condition, instead of a cue display, the memory probe display is shown immediately after the noise mask, and simultaneously with the probe array

The trial layout is visualized in Figure 2. Each trial began with a blue fixation dot (0.5 dva [degrees visual angle]), centered on the screen on a dark-gray background and 12 light-gray placeholder dots (0.23 dva), indicating the location of the upcoming memory items. Upon pressing the space bar, the fixation dot turned red and after 500 ms, 12 red bars were presented as memory stimuli for 500 ms, randomly oriented in one of four possible orientations (0*°*, 45*°*, 90*°*, 135*°*). Exact stimulus positions can be extracted from the second upper panel of Fig. 2.

In the retro-cue condition, after a 1,000-ms blank interval, during which only the fixation and placeholder dots were visible, a cue consisting of a white circle (0.6 dva) was presented for 250 ms at one of the 12 stimulus locations, followed by another 1,000-ms blank interval, followed by the memory probe display. The retro-cue was always valid. In the post-cue condition, the memory display was presented immediately after the first 1,000-ms blank interval.

The memory probe display consisted of 12 red, randomly oriented bars with a white circle (0.6 dva) indicating the test item, which either had the same orientation as in the memory stimulus (50% of trials) or was rotated by 90°. Participants pressed one of two keys to indicate whether or not the test item had changed. The memory display remained visible until participants gave a response.

## Procedure

Participants took part in eight experimental sessions of 1.5 hr each. The first five sessions took place Monday through Friday of the first week and the last three sessions Monday through Wednesday of the second week. At the beginning of the first session, participants were familiarized with the task during the course of 30 instruction trials (15 per condition), which were not included in the analysis. Instruction trials had a set size of eight (instead of 12) and verbal instructions were provided by the experimenter. Every eight trials contained four post-cue and four retro-cue trials presented in random order.

Participants were instructed to fixate on the dot in the center for the duration of a trial until they gave a response. If a participant’s gaze deviated 2.5 dva from the central fixation dot, or if the participant blinked, the fixation dot turned into a blue “x,” and the trial was aborted and repeated later. This was done to ensure that observers maintained central fixation during the crucial stages of the memory paradigm.

To ensure that the stimuli elicited no retinal afterimages, the gray value of the screen background was calibrated to be perceptually isoluminant to the memory stimuli for every participant at the start of each session. This was done by means of heterochromatic flicker photometry (Ives, 1912; Wagner & Boynton, 1972). In addition, a full-screen color noise mask was presented for 60 ms (six noise frames of 10 ms) following the offset of the memory stimuli. Prior to the experiment, participants chose one of six cartoon characters which would “evolve” into another version of that character for each additional 5% accuracy the participants achieved. This was implemented to keep participants motivated and was well appreciated. After every 20 trials, a progress graph was displayed that plotted correct and incorrect responses over time, with diagonal lines indicating participant’s cumulative accuracy during the current experimental session. We have included a depiction of a progress screen in the online materials.

Participants received auditory feedback after each response, indicating a correct (low-pitched sound; 300 Hz) or incorrect response (high-pitched sound; 500 Hz). Auditory feedback was provided to facilitate task learning in the observers. Observers were encouraged to take short breaks every 10–15 minutes, after which the eye tracker was recalibrated.

Several differences between the present study and the paradigm used in Sligte et al. (2008) exist. Firstly, we use a noise mask rather than a light flash to mask retinal afterimages because we found that this type of mask is less intrusive for observers than a bright flash of light after every trial, considering that they perform the task for 12 hours in total. The noise mask is as effective as a light flash, and, like the light flash, does not interfere with reportable sensory memory representations. Indeed, previous research has established that representations that can be retrieved via the retro-cue are only susceptible to masks that resemble the memory stimulus (Pinto et al., 2013). Secondly, we use 12 items instead of varying set sizes. We test a specific prediction that follows from the findings of Sligte et al. (2008): If retro-cues can access a memory system with a capacity of at least 16 items given a set size of 32, then, with enough practice, observers should reach ceiling performance with a set size of 12. This set size was chosen to be considerably above the “magic number four” (Cowan, 2001), while remaining well below the largest item-capacity observed in Sligte et al. (2008). Thirdly, while Sligte et al. used a block design for post-cue and retro-cue conditions, we interleaved trials in order to observe performance in both conditions evolve continuously over time (see Setup and Stimuli). Importantly, these differences affect both conditions equally.

### Data analysis

In the figures and analyses, trial number indicates “trial time,” in which both conditions are assumed to proceed in parallel. Thus, trial time 100 is the 100th successive post-cue trial or the 100th successive retro-cue trial. Ten observers performed 42,550 trials in total, 21,275 per condition. Trials per observer and condition ranged between 1,587 and 2,502. Observers completed different amount of trials as they moved at different speeds. The analyses reported here were performed for trial time 1 to 1,587, which is the last trial time that contains data from all 10 observers.

**Fig. 3:**
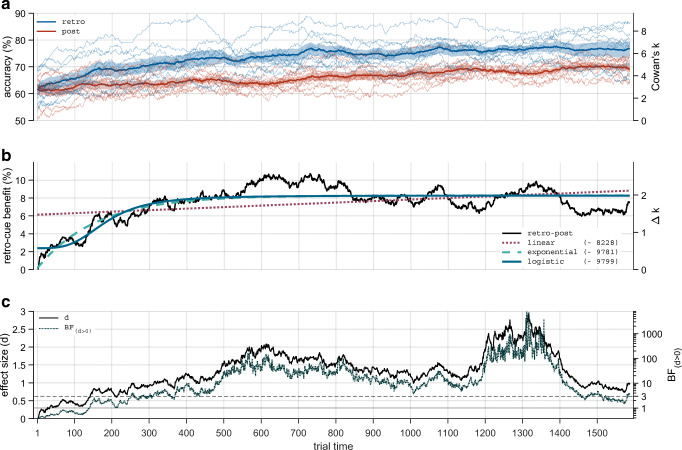
Main results for a moving average window size of 200 trials. Result graphs for a wide range of window sizes can be obtained in the online materials (osf.io/9xr82) and an animated version (gif) at (osf.io/wqz8g). **a** Accuracy in the post-cue and retro-cue conditions as moving averages. Thin lines indicate results from individual subjects. Thick lines indicate the estimated mean accuracy for each trial time. Shaded areas indicate estimated standard error. **b** Retro-cue benefit (retro-cue accuracy minus post-cue accuracy) and model fits. **c** Effect size and evidence for an effect size greater than zero for each trial time. The solid horizontal line indicates BF = 1 and the dashed horizontal line indicates BF = 3, a popular threshold for “evidence worth considering”

To estimate performance (probability of a correct response) over time, a continuous accuracy score per trial (percent correct) was obtained by computing a moving average over binary performance trial data (correct/incorrect) from each observer. The centered moving average window was shrunk when reaching the start and end of the data array, such that the filtered signal at, for example, Trial Time 20 represents the average of 20 trials to the left and 20 trials to the right. This estimation, however, is dependent on the window size used in the moving average, which acts as a low-pass filter. The signal is therefore a function of window size: small window sizes reveal high-frequency fluctuations in performance, whereas large window sizes reveal low-frequency fluctuations in performance. Since the frequency of relevant changes in performance over time (learning curves) is undetermined, we present analyses for different window sizes in the online materials. Very small window sizes mostly pick up on noise (fast performance fluctuations are most likely related to variations in attention), and very large window sizes provide no additional benefit in noise reduction. The primary result of the present paper (i.e., the qualitative development of the retro-cue benefit over time) was not dependent on window size. However, the estimated time point at which the retro-cue benefit first reliably emerged, and the estimated time point at which it plateaued did vary as a function of window size. As optimal visualization duals optimal analysis, and for simplicity, we present average performance per condition in Fig. 3 and report model comparisons for Window Size 200 only and provide results for all window sizes in the online materials.

The results of the moving window averaging approach can be interpreted by considering the filtered data as representing one participant’s success rate at a given trial time in the experiment, when averaging over a number of trials equal to window size. For instance, in our data, Observer 1 has an accuracy of 0.81 at Trial 200 when using a moving average window size of 100. This means that if the participant performs 100 trials in an experiment after training for 150 trials, we expect a resulting percentage correct of 81% for those trials. Note that the continuous accuracy values from trial time one to the trial time that is equal to one half of the window size are subject to shrinking window sizes (see previous paragraph), and thus datapoints at the very beginning of the array should be interpreted with caution. The same is true for the datapoints at the end of the array.

Using the filtered data from the 10 observers, Bayesian estimation performed in JAGS (Plummer, 2003), using the *matjags* MATLAB interface (Steyvers, 2011), produced group means and variance for each trial and condition. These group means (thick lines in Fig. 3a) can be considered a hyperparameter of the population distribution which is generating these success rates. Cowan’s *k* was approximated based on accuracy scores using Equation 1:

1 $$ k=\left(2\times \mathrm{accuracy}-1\right)\times \mathrm{set}\ \mathrm{size}. $$

The retro-cue benefit was subsequently calculated by subtracting the estimated group means in the post-cue condition from those in the retro-cue condition. A linear, exponential, and logistic function (see Table 2; Equations 4–6) was fitted on the retro-cue benefit through least squares regression in MATLAB. The Bayesian information criterion (BIC; Schwarz, 1978) of the fits were compared  to determine the most likely model underlying the development of the retro-cue benefit over time. BIC was calculated using Equation 2, where *LL* is the log-likelihood of the model, *N* is the number of datapoints, and *k* is the number of parameters in the model. Larger (negative) BIC’s indicate a better model fit:

**Table 2:** Model equations

Equation	Model	*f*(*x*_*i*_)=	Free parameters	
(4)	Linear	*m* · *x*_*i*_ + *c*	*m*, *c*	slope, intercept
(5)	Exponential	$a\cdotp {e}^{-k\cdotp {x}_i}+c$	*k*, *a*, *c*	exponent, scaling factor, intercept
(6)	Logistic	$$ \frac{\begin{array}{c}\ \\ {}\ \\ {}{l}_1+\left({l}_0-{l}_1\right)\end{array}}{\begin{array}{c}1+{\left(\ \frac{x_i}{x0}\ \right)}^k\\ {}\ \end{array}} $$	*k*, *x*0, *l*_0_, *l*_1_	exponent, inflection point, lower asymptote, upper asymptote

2 $$ \mathrm{BIC}=-2\times LL+\ln (N)\times k. $$

In addition, we estimated the standardized effect size between conditions for each trial using Eq. (3) below, and quantified group level evidence as Bayes factors in favor of an effect size greater than zero using a Student’s *t* prior with *df* = 1 (i.e., a standard Cauchy prior; Lee & Wagenmakers, 2014, p. 124).

3 $$ d=\frac{\mu_1-{\mu}_2}{\sqrt{\frac{{\sigma_1}^2+{\sigma_2}^2\ }{2}}}. $$

We welcome the reuse of the data, stimulus materials, and analysis scripts, which are available at osf.io/9xr82 and github.com/paulzerr/retrocues.

## Results

### Retro-cue benefit did not increase indefinitely with practice

Figure 3a–b clearly demonstrate that the retro-cue benefit did not continue to increase over time after its initial emergence. A logistic function (BIC = −9,799) provided a better model fit than a linear (BIC = −8,228), or exponential function (BIC = −9,781). This result also becomes immediately apparent to the “naked eye” when observing Fig. 3b. The logistic model suggests that participants were initially not better at retro-cue trials than at post-cue trials, then rapidly learned to use the retro-cue, and the retro-cue benefit subsequently reached a stable level. The logistic and exponential models converged on a maximum difference in capacity of ∆*k* = 2 items, or a difference in accuracy of 8.3% (i.e., the asymptote of the functions).

When removing one subject at random from the analysis, BIC’s for linear fits range from −7,793 to −8,597, exponential fits range from −9,056 to −10,056, and logistic fits range from −9,075 to −10,140. This means that in each permutation, the exponential or logistic model (both indicating that the retro-cue benefit reaches a plateau) is preferred over the linear model (indicating a continuous increase). The logistic model is preferred over the exponential model in 7 out of 10 permutations.

In the first derivative of the logistic fit (i.e., the function of change in the magnitude of the retro-cue benefit) the point when the retro-cue benefit changes less than 0.01% per trial (i.e., less than 1% per 100 trials) is reached at trial time 291, the point when the retro-cue benefit changes less than 0.001% per trial (i.e., less than 0.1% per 100 trials) is reached at trial time 535. This provides an indication for when the retro-cue benefit stops to increase in a meaningful way in the present data.

**Fig. 4:**
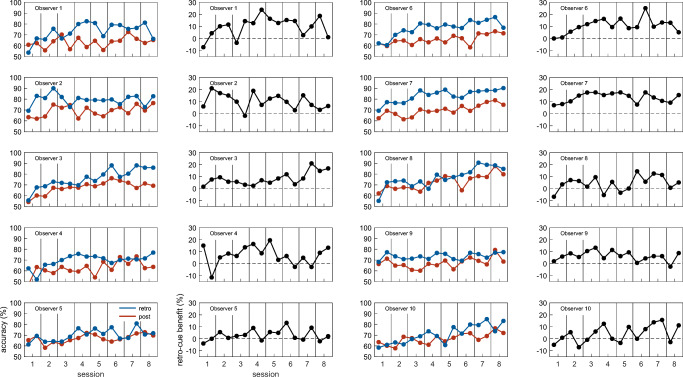
Individual observer data as percent correct, split into two bins per experimental session. All observers demonstrated a retro-cue benefit after some time

Figure 4 displays data of individual observers as percent correct. Exploratory analyses revealed a mean change of the retro-cue benefit between the second half of one session and the first half of the following session of 0.002 (or 0.2% accuracy) across participants (*SE* = 0.01), indicating transfer of learning between sessions but no evidence for sleep consolidation effects on the group level.

### Emergence of the retro-cue benefit

As described in the Data Analysis section, the result of the moving average transformation plotted in Fig. 3a has a direct and meaningful interpretation: An accuracy value at trial *N* represents the proportion of correct trials an observer obtained across a number of trials that is equal to the window size used after *N* − (window size / 2) trials of training. This translates to the level of evidence a researcher might obtain after a given amount of training and when using a given number of trials per condition. As can be seen in Fig. 3c, evidence reliably crosses the popular BF = 3 threshold around trial time 300. Result patterns that are obtained when including different amounts of trials per condition can be observed in the online materials.

### Accuracy continued to increase in both conditions

Notably, average accuracy continued to increase in both the post-cue and the retro-cue conditions (see Fig. 3a). An exploratory analysis indicated that linear models fitted from trial 500 to 1,587 revealed slopes greater than zero (m_post_ = 6.2 × 10^-5^, 95% CI [6.0 × 10^-5^, 6.3 × 10^-5^], or 0.62% increase per 100 trials; m_retro_ = 3.7 × 10^-5^, 95% CI [3.5 × 10^-5^, 3.8 × 10^-5^], or 0.37% increase per 100 trials). This means that, at 1,587 trials, observers’ average performance was still increasing in both the post-cue and the retro-cue conditions. A linear model fitted from trial 500 to 1,587 for the difference between conditions (i.e., the retro-cue benefit) revealed a slightly negative slope (m_retro-post_ = −2.5 × 10^-5^, 95% CI [−2.7 × 10^-5^, −2.3 × 10^-5^], or a 0.25% decrease per 100 trials). This provides further evidence that retro-cue benefits did not continue to increase indefinitely, and if anything, slightly decreased over time with excessive training.

## Discussion

Retro-cues allow observers to reallocate attention within the contents of VSTM to prioritize an item prior to a memory task and increase its probability of recall. While previous studies reported performance averaged across time, we investigated the learning curves of 10 human observers performing a retro-cue change-detection task for 12 hours each. We compared the development of accuracy over time between retro-cue and post-cue conditions to discern qualitative and quantitative differences in learning rates, which could reveal differences in VSTM mechanisms involved in the two conditions.

### No evidence that retro-cues access a separate high-capacity store

If retro-cues enable access to a high-capacity VSTM store that is able to hold 16 items or more (Sligte et al., 2008), then with enough practice, observers should be able to reach near ceiling performance, given our current set size of 12 items. However, in our experiment, the difference in performance between the retro-cue and post-cue conditions quickly stabilized at about *∆k* = 2 items (or a difference of 8.3% accuracy). Specifically, this retro-cue benefit plateaued after a few hundred trials. At that point, performance had reached a capacity of *k* = 4 in the post-cue condition and a capacity of *k* = 6 in the retro-cue condition. This result does not support the hypothesis that retro-cues allow observers to access high-capacity sensory memory or LTM (H_1_), as it would predict that in a memory task with a set size of 12 items, the retro-cue benefit continues to increase until observers reach an estimated working memory capacity of close to *k* = 12 items because sensory memory and LTM should not be subject to the same resource bottleneck (i.e., attention) as VWM. Instead, the data show that the retro-cue consistently adds two items to stable working memory capacity. This result favors the hypothesis that retro-cues allow observers to access a portion of VSTM that received sufficient attention to be encoded into capacity-limited VWM, but not enough attention to be reportable in a change-detection task without retro-cues (H_2_). This implies that VWM contains more information than estimated in a task without retro-cues—or, in other words, that some items in this memory store are encoded less robustly and can be prioritized retrospectively.

On the other hand, it has been observed in neuroimaging results that unattended items that could not be decoded during the retention interval, were once again decodable after an attentional shift induced by the retro-cue (e.g., Sahan et al., 2019) and it has been suggested that objects in VSTM can exist in activity-silent states (e.g., Stokes, 2015; Wolff et al., 2015; Wolff et al., 2017). Such a qualitatively different neural storage implementation would support the idea of a categorically different memory state for attended and unattended items, as also proposed by the multiple-store hypothesis. In response to this interpretation, however, it has been suggested that unattended memory items are not stored in a categorically different way, but that the neural activity elicited by unattended items persists, albeit with an amplitude too small to be picked up by the imaging technique (e.g., Rademaker & Serences, 2017; Schneegans & Bays, 2017). The present results also speak more strongly for quantitatively, rather than qualitatively, different states within VWM.

Behaviorally, Makovski (2012) also concluded support for the single-store hypothesis from the finding that a retro-cue benefit exists even when the retro-cue is displayed after visual interference. However, the authors used a set size of only four items, which may be insufficient to investigate a high-capacity system. Further evidence for a single store comes from Robinson and Irwin (2019), who used a state-trace analysis to assess the dimensionality of VSTM and concluded that the results were more parsimonious with the single-store hypothesis.

The activated long-term memory (LTM) hypothesis of working memory (Cantor & Engle, 1993; Oberauer, 2002; Öztekin, Davachi, & McElree, 2010; Ruchkin, Grafman, Cameron, & Berndt, 2003) describes a three-state model of working memory: (1) activated LTM, which can keep task-relevant information such as feature spaces available and is not capacity or attention limited; (2) a “region of direct access,” which is closely related to traditional VWM, capacity limited, and represents a “broad focus” of attention; and (3) a single item in the focus of attention. Within this framework, the present results are more parsimonious with the view that retro-cues prioritize (focus attention on) one item from the broadly attended “region of direct access” and protect it from interference (i.e., H_2_), rather than moving information from LTM into the “region of direct access” (i.e., H_1_).

In conclusion, while there remains the possibility that some objects in VSTM are represented by a qualitatively different mechanism and that the visual system contains high-capacity information stores, the present findings suggest that retro-cues operate within VWM by redistributing attentional resources and prioritizing a relevant object (Nobre et al., 2003; Souza & Oberauer, 2016). This implies that retro-cue paradigms (or for that matter partial-report paradigms) may not be suitable to investigate the existence or functionality of high-capacity memory stores. Other methodologies, such as rapid serial visual presentation paradigms (Endress & Potter, 2014), may yet be able to reveal and investigate high-capacity VSTM. Our results further suggest that VWM has a larger capacity than estimated in change-detection tasks without retro-cues.

### Continuous performance increase in both conditions

While the retro-cue benefit remained stable, accuracy continued to increase in both conditions throughout the experiment and this increase was still present after 12 hours spent on the task. While this parallel increase in both conditions provides further support for the single-store hypothesis, it is also surprising. Working memory capacity is often considered to be a stable trait of an observer (Xu, Adam, Fang, & Vogel, 2018), and is correlated with several personality traits, such as fluid intelligence (e.g., Feldman, Tugade, & Engle, 2004). Since different observers respond differently to the same amount of practice, this is especially problematic when the estimated capacity in a change-detection task is taken as an absolute measure (e.g., trait of an individual), such as in correlational (e.g., Shipstead, Redick, Hicks, & Engle, 2012) and developmental studies (e.g., Riggs, McTaggart, Simpson, & Freeman, 2006; Simmering, 2012), rather than a relative measure (e.g., within-subjects comparisons). In addition, most studies investigating working memory capacity report performance collapsed across time. Meta-analyses cannot adequately compare such studies without taking into account the amount of practice observers received (either before or during the experiment).

Furthermore, it is highly unlikely that an increase in performance over time represents an increase in actual memory capacity, at least not within 12 hours. One possibility is that observers learned to distribute their attention more evenly across the memory items during encoding. Observers may also have been relieved of crowding effects over time (Yashar, Chen, & Carrasco, 2015). Matsukura and Hollingworth (2011) also pointed to changes in processes unrelated to memory capacity (e.g., the efficiency of perceptual processing, memory encoding, maintenance, comparison processes, or involvement of long-term memory as potential explanations for a continuous increase in performance in change-detection tasks). They tested the latter hypothesis in a control experiment by comparing performance between using bars with either two or four possible orientations, under the assumption that relations between items (i.e., chunking) would be more easily stored when using two possible orientations (horizontal and vertical). They found no difference between these conditions, suggesting that context effects do not explain high-capacity estimates. Sligte et al. (2008) also tested this possibility and found no difference in performance when a single item or all items were shown in the memory task display. However, other researchers did find evidence that ensemble representations may facilitate memory processes and indeed may inflate capacity estimates, albeit for complex objects (Brady & Alvarez, 2015). As such, it remains unclear what factors drive the continuous increase in performance over time that we observed in the current experiment.

The finding that performance continuously increased in the post-cue condition casts doubt on the usefulness of change-detection tasks in estimating VWM capacity of an observer as it may not be possible to generalize across experiments that are employing different amounts of practice as well as difficulties when investigating individual differences. More generally, an experimental method that yields an ever-increasing measure of VWM capacity may not be well suited for measuring actual VWM capacity, which is typically considered stable over time.

### Practice does not account for previously observed, very high Cowan’s *k* estimates

It is important to point out that the continuous increase in performance, described above, was observed in the post-cue condition as well as the retro-cue condition. Matsukura and Hollingworth (2011) also reported a continuous increase in performance when two observers practiced a retro-cue change-detection task over the course of 80 minutes. From this, they concluded that extensive practice could account for the large performance differences between post-cue and retro-cue conditions observed by Sligte et al. (2008). However, Matsukura and Hollingworth (2011) did not include a post-cue control condition to ascertain that the increase in performance was specific to the retro-cue condition. The present data show that these practice effects are not specific to the retro-cue condition, and therefore cannot account for the retro-cue benefit and high VWM item capacities. More specifically, Fig. 3b of the present paper clearly shows that increases in performance that are specific to the retro-cue effect conclude after a few hundred trials, as retro-cue benefits no longer increase (while performance in both conditions continues to increase in parallel). In other words, continued practice in the task increases performance in both conditions and does not selectively increase the retro-cue benefit. Matsukura and Hollingworth (2011) further critiqued the multiple-store hypothesis by suggesting that capacity estimates drop from 16 to five–seven items when two instead of four possible orientations are used. However, these lower capacity estimates were observed in experiments with a set size of only eight items. Thus, the extremely high item-capacity estimates observed by Sligte et al. (2008) remain intriguing and will require further investigation to be reconciled with the VSTM literature, including our study.

### The limits of the magic number four and implications for item limits in VWM

The item-based capacity measure Cowan’s *k* (Cowan, 2001) is intended to provide an estimate of the number of items that an observer can maintain in memory irrespective of set size. Indeed, *k* is stable across set sizes (Rouder et al., 2011) and even across experiments (Xu et al., 2018) when no retro-cues are employed. However, with retro-cues, *k* (and the retro-cue benefit when expressed in *k*) does scale with set size, such as in the study by Sligte et al. (2008). This was also reported by Souza et al. (2014), albeit with a smaller set size of one to eight items. This means that when not using retro-cues, *k* underestimates the memory capacity of an observer, because more fragile, less attended memory items (which were nonetheless encoded into VWM), are not captured by the memory task. On the other hand, when retro-cues are employed, *k* is dependent on set size. While *k* seems to offer an intuitive interpretation of capacity as an item limit, these considerations call for caution in interpreting this metric as a proxy of VWM capacity.

These limitations likely stem from the assumption inherent in *k* that the underlying resource in VWM is composed of discrete slots. This view finds support in the following observation: When the results by Sligte et al. (2008; taken from Fig. 2, p. 3) are back-transformed from Cowan’s *k* to percentage correct, performance for Set Sizes 4, 8, 16, and 32 dropped from about 88% to 74%, 62% and 60% in the post-cue condition, while performance in the retro-cue condition dropped from about 100% to 94%, 84% and 75%. Thus, the observation that the retro-cue benefit (and therefore VWM capacity) scales with set size no longer holds when the differences between conditions are expressed as percentage correct: 12%, 20%, 22%, and 15%. Percentage correct can be interpreted as the average probability of any item to be recalled. A similar retro-cue benefit to recall probability across different set sizes suggests that retro-cues enable access to a fixed amount of a flexible resource (attention or the information carrying capacity of the VWM system; e.g., Bays & Husain, 2008; Schneegans & Bays, 2016) rather than a fixed number of item slots (e.g., Luck & Vogel, 2008). This view is also more supported by our present findings, which suggest that retro-cues redistribute attention within VWM. For a discussion of retro-cue benefits in relation to discrete slot (e.g., Cowan’s *k*) and flexible-resource models of capacity-limits in VWM that is beyond the scope of the present paper, see Souza et al. (2014).

### Large retro-cue benefits necessitate long practice sessions

An important conclusion from the present data is that observing large and reliable retro-cue benefits requires generous practice before experimental data is collected. Large individual differences exist in the speed and extent of learning to utilize the retro-cue, and a long training session before recording experimental data is highly recommended to avoid false nulls, and especially if it is not the retro-cue benefit itself that is of interest, but a modulation thereof. Generally, it should be avoided to collect experimental data on an effect that still continues to increase during the experiment session, especially if the rate of increase might differ between observers. Based on the current results, a good rule of thumb would be to include about 500 trials of practice before collecting experimental data for maximal effects.

## Summary

Our results suggest that retro-cues operate within the contents of VWM and do not provide evidence that retro-cues access a high-capacity memory store that is independent of the resource limitation of VWM (such as sensory memory or LTM). Additionally, the present data cast doubt on the usefulness of change-detection tasks and Cowan’s *k* in estimating VWM capacity. Finally, we suggest that studies using change-detection retro-cue experiments employ generous amounts of practice trials before collecting experimental data.
